# Patients’ views and experiences on the use and safety of directly acting oral anticoagulants: a qualitative study

**DOI:** 10.1186/s40545-023-00563-y

**Published:** 2023-05-01

**Authors:** Abdulrhman Al Rowily, Mohamed A. Baraka, Mohammed H. Abutaleb, Aliah M. Alhayyan, Nouf Aloudah, Zahraa Jalal, Vibhu Paudyal

**Affiliations:** 1 School of Pharmacy, Institute of Clinical Sciences, College of Medical and Dental Sciences, Sir Robert Aitken Institute for Medical Research, University of Birmingham, Birmingham, B15 2TT UK; 2 Pharmaceutical Care Department, King Fahad Military Medical Complex (KFMMC), Medical Department, Ministry of Defence, Dhahran, Saudi Arabia; 3 College of Pharmacy, Al Ain University, Al Ain, United Arab Emirates; 4 Pharmaceutical Care Department, King Fahd Central Hospital, Jazan Health Affairs, Ministry of Health, Jazan, Saudi Arabia; 5 Third Health Cluster, AL-Diriyah Hospital, Riyadh, Saudi Arabia; 6 Department of Clinical Pharmacy, College of Pharmacy, King Saud University, Riyadh, Saudi Arabia

**Keywords:** Direct oral anticoagulants, DOACs, Medication errors, Patients, Patient experience, Qualitative research, Interviews

## Abstract

**Background:**

Direct oral anticoagulants (DOACs) are considered high-risk medications and used to prevent thromboembolic events and stroke. This study aimed to examine patients’ views and experiences of DOACs use and factors that can promote safety associated with DOACs.

**Methods:**

In-depth interviews were conducted with adult patients who had been prescribed DOACs, identified and invited by local collaborators in three different tertiary care hospitals in Saudi Arabia. A topic guide developed based on was used to inform the interview. Data were analysed thematically.

**Results:**

Data saturation was achieved by the ninth participants. Three major themes were identified: (1) factors affecting DOAC's safety from the patients view; (2) barriers to adherence to DOACs and (3) strategies to promote the safety of DOACs. Lack of knowledge of DOACs, using inappropriate sources of information, lack of communication with HCPs, difficulty in having access to DOACs and lack of monitoring were the main factors affecting the safe use of DOACs. Unavailability of the drugs and difficulty in timely getting to hospitals affected adherence. Patients acknowledged difficulties communicating with healthcare professionals, timely access to anticoagulation clinics and in obtaining their DOACs on time.

**Conclusions:**

There is a need to develop and evaluate theory-based interventions to promote patient knowledge, understanding and shared decision-making to optimise DOACs use and improve their safety.

## Introduction

Direct oral anticoagulants (DOACs) are extensively prescribed for prevention of stroke and thromboembolism in patients with non-valvular atrial fibrillation (NVAF). NVAF is becoming highly prevalent in the population with risk estimates reaching about 1 in 3 in white individuals and 1 in 5 for black individuals over the age of 50 [1]. Moreover, patients with these conditions are often associated with long-term co-morbidity, polypharmacy, frequent hospitalisation, reduced quality of life and poorer physical function, thereby increasing likelihood of errors [2, 3].

The safety and effectiveness of DOACs is highly dependent on safe prescribing as well as the ability of patients to manage and take the medicines. Medication errors associated with DOACs pose significant health risks to patients. A recent systematic review and meta-analysis conducted by the study researcher showed that 20% of patients prescribed DOACs experienced some sort of prescribing error [4]. Another study reported 62% of all DOACs errors caused patient harm, of which 5.2% caused severe harm and 0.8% were fatal errors [5]. Most of the fatal errors were due to wrong dose errors, while the most severe harm errors were due to medication omissions. In another study, DOACs were most commonly associated with severe harm, including 25% of all fatal errors, making it the most harmful therapeutic area in the reported incidents [5]. Other studies have shown that medication errors associated with DOAC’s resulted in high mortality and morbidity and cause a long stay in hospital [6, 7]. A retrospective study by Piazza et al. identified that the medication errors were the common root cause in 40% of anticoagulation-related adverse events [8].

Suggested strategies to improve medication safety and prevention of medication errors from occurring include the provision of clinical pharmacist within the clinical team. In addition, it is important to strengthen the communication between healthcare professionals and patients [9]. Despite high prevalence of errors associated with DOACs, there is a dearth of research looking into patient perspectives on the use of DOACs from the patient perspectives.

Patient participation in research is considered a key component for enhancing safety and quality of healthcare services. It is important for patients to be allowed to make informed decisions about their treatment. Lack of patient engagement in the prevention and management of cerebrovascular disorders has previously been previously flagged [10].

Therefore, this study aimed to examine patients’ views and experiences of DOACs use and factors that can promote safety associated with DOACs.

## Materials and methods

### Participants

In-depth interviews were conducted with adult patients recruited from tertiary care hospitals in Riyadh, Dhahran and Jazan regions of Saudi Arabia. Online videoconferencing was used. One tertiary hospital in each of the three Saudi regions were chosen. Hospitals were eligible to participate in the study based on the presence of a cardiology department with at least 2 consultant cardiologists. Purposive sampling was undertaken for the recruitment of participants. A maximum variation sample was undertaken including those taking high/low DOAC's use years, and sex. Patients were interviewed until data saturation was achieved and no new code emerged. Saturation of the data collection was achieved after 7 interviews with patients and further two interviews were conducted to confirm it.

### Procedure

An invitation e-mail was sent to the anticoagulation clinic through cardiology department in the Hospitals for participants' recruitment. In each hospital, a local collaborator, identified the patients from the anticoagulant clinic list and contacted eligible participants. The collaborator explained the study objectives and provided the Arabic language information sheet to the participants during a face-to-face meeting and if agreed, the consent was signed by the collaborator.

Ethical approval was obtained from the University of Birmingham Research Ethics Committee (ERN 20-0551). Moreover, three ethical approval was obtained from the three hospitals: National Guard Hospital in Riyadh (SP20/212/R), King Fahad Military Medical Complex in Dhahran (AFHER-IRB-2020-015), and King Fahad Central Hospital in Jazan (164/2020). Prior enrolment in the study, all participants was endorsed about the study and signed an informed consent form.

### Materials

A topic guide was developed by the main researcher (AA) after based on the literature [4, 11]. Questions were focused on the experience in taking DOACs, concerns raised during their use, the perceived level of knowledge they have about these drugs, and the perceived safety culture. Specific questions about the factors associated with errors and their prevention were also asked. The questions were revised by the research team and the collaborators including physicians and pharmacists who included experts in both qualitative research and DOAC's use. The interviews were conducted in Arabic, recorded, and transcribed to Arabic language and translated to English language in final stage of analysis when emerged themes and subthemes and choosing the quotes.

### Data analysis

The interview data were systematically analysed using a thematic content approach using the software program MAXQDA Analytics Pro 2020 (VERBI Software). Each transcript was independently analysed by two authors (AA, NA), a third author checked both versions (VP) for discrepancies. Disagreements were resolved by discussions. As the semi-structured interviews progressed, data were analysed after each interview to develop initial codes and to identify important and new emerging information. The final themes and related quotations were translated to English language.

Semi-structured interviews were conducted virtually using Zoom videoconferencing as a platform due to limitations caused by the Covid-19 pandemic. The study was undertaken from September 2021 to end of November 2021 and interviews lasted between 30 to 45 min in duration.

### Rigour and trustworthiness

To improve rigour and trustworthiness, each interview was ended by a summary to be validated by the participants and to check any ambiguity. After each interview the two researchers (AA and NA) met and reflected on the information received. Memos (such as reporting interviewee’s facial expressions or hesitancy to answer certain questions adequately) and journaling were recorded during the interviews and utilised throughout data collection and analysis using MAXQDA memos. Data from interviews were kept secure in computers with a secured password. This study was reported in accordance with the Consolidated Criteria for Reporting Qualitative Research (COREQ).

## Results

A total of nine participants were recruited. Most of the patients were male (*n* = 6) and had a Bachelor of Science as their degree (*n* = 5). Table 1 summarises participant characteristics.

**Table 1:** Participants’ characteristics (*n* = 9)

Participants characteristics	Patients (*n* = 9)
Gender	
Female	3
Male	6
Experience of DOACs uses	
< 6 months	4
6–12 months	2
1–2 years	1
2–3 years	1
> 3 years	1
Education	
Undergraduate degree	5
Complete school aged 18	3
Not disclosed	1
Current job title	
Fully retired from work	3
Unemployed	3
Not disclosed	3

**Fig. 1:**
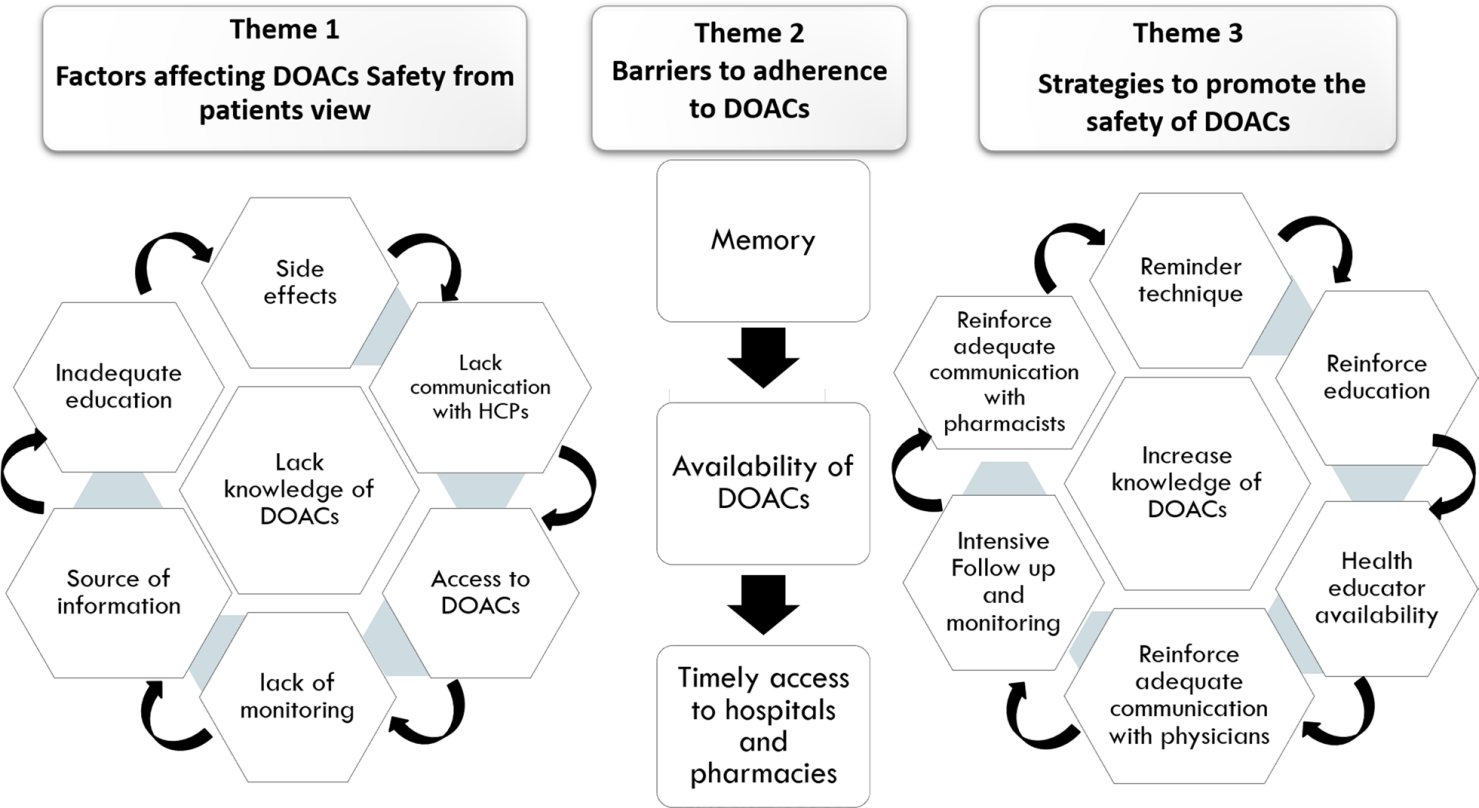
An overview of themes and subthemes that emerged during data analysis

The analysis of the interviews identified three themes: (1) factors affecting DOAC's safety from the patients view; (2) barriers to adherence to DOACs and (3) strategies to promote the safety of DOACs. Figure 1 represents an overview of themes and subthemes that emerged during data analysis.

### Theme 1: Factors affecting DOACs safety from patients view

#### Knowledge

##### Lack of knowledge on DOACs

Most participants described having inadequate knowledge about dose, drug–drug interactions, duration, and side effects. They described lack of adequate time spent with the doctors about answering these queries. Many doctors often referred patients to pharmacy to request further information about the prescribed DOACs.

“*….I am not aware of all the information regarding the medication prescribed by the doctor? What I know about him is that he only “blood thinner”, And when I am in the clinic with the doctor, he does not give me enough time to find out about the disease and the treatment. He only tells me to go to the pharmacy to take the medicine, and I have other patients, waiting their appointment please*.” *patient 1 (Riyadh)*

Many described lack of opportunities and busy schedule in the clinics as well as in pharmacies that prevented patients obtaining the information that they needed.

*“In fact, it is difficult to communicate. I tried to talk to him and explain more to me on the grounds that it was the first time I took the medicine. I talk about how I take medicine. He said to me: You use the medicine as it is written on the prescription, and when I went to the pharmacy they gave me the medicine, but I went back to the clinic asking the doctor. The clinic was crowded, and I was unable to communicate with him” patient 3 (Dammam)*

Other patient participants reported that pharmacists do not offer adequate time to counsel patients and often refer patients to the instructions in the patient information leaflet.

*“…When I go to the pharmacy to dispense the prescription, the pharmacist dispenses the medications to me and does not explain anything he said all instruction written in the label. as you know the pharmacy is crowded patients, just pack a bag, and go home” patient 3 (Dammam)*

#### Source of information

Most participants agreed that healthcare professionals only rarely provided them with educational information about DOACs. They described that they often had to rely on relatives, friends or use an untrusted site accessed through Google to find information regarding DOACs.

*“…. Of course, this medicine was previously taken by one of my relatives, and frankly, I always ask him about the medicine, is taking two doses better than one? When I gave my mother the wrong dose, it caused her to bleed for about five days, and she is in the intensive care unit in the hospital; after this case; I mean I learn it must be very careful with these drugs. If they exceed the normal limit or the required limit, they will have more harm than good. Therefore, in case of forgetfulness or doubt, it is preferable to ask the doctor and pharmacist, or use a leaflet of drug, or enter the Internet to give you general information” patient 2 (Riyadh)*

#### Side effects

Most participants described being unaware of the side effects including very common side effects such as bleeding. Yet again, lack of counselling from healthcare professionals was described.

*“Yes, I have side effects while taking the medicine, so I decided to stop the medicine. The first thing I felt was a pain in the knees, so it is, and I woke up from sleep, my mouth and gums were a little bloody. In fact, I felt worried, as the doctor did not tell me about the side effects of the drug” patient 1 (Riyadh)*

#### Communication

##### Lack communication with healthcare professionals

Lack of communication with healthcare professionals (HCP) was deemed a key factor compromising patient safety. Many patients indicated the physician did not engage patients in informed decision-making for choosing DOACs anticoagulants.

*“Frankly, I am like many other patients, had many diseases such as clots, diabetes, hypertension, and high cholesterol. So sometimes I neglect the use of medicines because they are many and I miss monitor them especially since I live alone and another thing the doctor not share us when choose the drug to make a decision what is suitable for me and what available drug or frequency of dose once or twice daily.” patient 6 (Riyadh)*

Lack of information often led the patients not knowing how to manage their medicines daily.

*“...Sometimes it is very difficult to communicate with the doctor or pharmacist, especially if I did not take the medicine for example if I forgotten. I do not know whether to take the medicine immediately or wait for the next day at the same time of the dose” patient 3 (Dammam)*

#### Access to DOACs

Some patients indicated that the lack of drug delivery services in the hospital affected the accessibility to medications and led to patients not receiving their medicines. For instance, during the Covid-19 pandemic transportation was deemed challenging.

*“Yes, the reason is that the hospital is far from housing, and the hospital does not have drug delivery services from the pharmacy if we need refill drug, and we were afraid to go to the hospital to refill medication, especially during the times of the Corona pandemic.” patient 7 (Dammam)*

#### Monitoring

Lack of monitoring especially in the anticoagulation clinic was deemed a key factor compromising patient safety. Many patients described difficulty obtaining appointments in anticoagulation clinic. Other participants reported not attending anticoagulation clinic due to their own carelessness.

*“...Sometimes it is very difficult to attend an anticoagulation appointment with the doctor, especially if you forget the day that I have to attend the anticoagulation clinic and sometimes basically the doctor himself forgets to give us an appointment to follow up with him at the clinic” patient 3 (Dammam)*

### Theme 2: Barriers to DOACs adherence

Under this theme, participants described factors associated with adherence to DOACs and administration errors.

#### Memory

Some patients described as having been prescribed multiple medications. Due to polypharmacy, many patients would often forget to take their DOACs.

*“True, to be honest, a lot. I forget to use medicines because I suffer from high blood pressure, diabetes, and heart problems. I have more than 10 medicines that I use. I forget to take medicines, frankly.” patient 4 (Riyadh)*

#### Availability of DOACs

This was identified as a major barrier to adherence. One patient realised that if the drug was not available in the hospital, it would cause the patient to miss doses, and some patients reported that if the drug was not available in the pharmacy, the patient would feel dissatisfied. Dissatisfaction also expressed by many patients who expressed the drug's high costs when they wanted to purchase it.

*“The big problem with edoxaban the availability of drug. I really hope that it is available for free in government pharmacies because most of the time it is not available. The worst is that its price is a little expensive, and every month need one pack is required; For a period of thirty days, it contains 28 tablets and cost is 240 SAR; Sometimes it means in some people cannot pay this price, what I can do, especially if they more than 2 drug out of stock.” patient 5 (Riyadh)*

#### Timely access to hospitals and pharmacies

Some participants described difficulty accessing DOACs mainly due to barriers in timely getting to hospitals and dispensing pharmacies.

*“Patient’s daughter: Eliquis particularly rare if it is available in the hospital, due to what we spent a lot of time to go in the same hospital in the same area. However, the hospital is a far away from our town and we had some difficulties to reach the hospital.” patient 7 (Dammam)*

### Theme 3: Strategies to promote DOACs safety

Patients’ participants suggested numerous ideas to help minimise errors related to the use of DOACs.

#### Reminders

##### Use the medication box to remember/mobile alarm/caregivers

In order to reduce suboptimal use and minimise administration errors, patients reported using multicompartmental compliance aids and digital app reminders. These were particularly deemed effective where carer support was not always available.

*“I am so careful I use it that I use the medication at the right time. So I made a schedule for myself and also a mobile alarm, because I have no one from the family to alert me, only my son is present and sometimes he alerts me and sometimes not, I have diabetes, and I have hypertension, so I set a schedule for me and when do I take the doses so that I do not forget to take them, in a paper I have a morning What should I take in the evening and what should I take in the evening?” patient 2 (Riyadh)*

#### Following advice from HCP

Some patients described that safe and effective use of DOACs requires patients to follow instructions provided by healthcare professionals.

*“The best way to safely use the drug so that there are no errors; The most important thing is to follow the doctor’s instructions no matter what, as well as the pharmacist’s question, you go and ask, in such and such, but it is always better and better to refer to the medical source, which is the doctor “patient 9 (Jazan)*

#### Education

##### Availability of a health educator in hospital

Some patient participants wished that there was a health educator in the hospitals that they could talk to about their medicines. Such health educators would fulfil the roles of education provider that they often expect from their doctors and pharmacists. Participants also referred to pharmacists not undertaking the roles of information providers.

*“.... I wish there is a clinic that educates about this medicine, frankly, the doctor and the pharmacist are always busy, I mean, they don't give me enough information. The doctor says, "Take this medicine first thing in the morning and go home, and the pharmacist doesn't explain enough because the label is on the medicine. I wish there was a health educator." He sits with me and teaches me, what should I do if I forget to take the medicine, what if I have certain symptoms, what should I do. And so on” patient 2 (Riyadh)*

*“We need to have a health educator after receiving the medicines explaining to us the reasons for using this medicine and the pros or cons of the medicine. Frankly, we have medical consultations for them with a special number, so I can call them. But this is not enough because sometimes they are not available.” patient 3 (Dammam)*

*“I stress that there should be a medical educator who sits with the patient, talks to the patient, and teaches him about what are the symptoms, if for example something happened to me or if I felt a certain symptom, what is should I and should not do it?” patient 4 (Riyadh)*

#### Intensive monitoring and follow-up

Regular follow-up to the clinic was identified as another strategy to promote optimal use of DOACs. They described that intensive follow-up can help identify patients at risk and medication errors associated with DOACs to mitigate them.

*“One of the good things and makes me feel comfortable going to the coagulation clinic, they give me an appointment every three months, about a year every three months. Then they put it every 6 months, then every eight months. On this visit, the doctor was checking an electrocardiogram and making sure of blood thinner tests I advise all patients to commit to visiting the liquidity clinic regularly” patient 6 (Riyadh)*

##### Reinforcing adequate communications with HCP

Patient participants stressed the need for adequate communication with health care providers outside clinic hours to answer, confirm and support the patient with any inquiries about safe use of medicines.

*“I hope there is a way to communicate with the doctor outside the coagulation clinic. This will make it easier for me to identify all my inquiries, especially if side effects occur. Also, with regard to medication, I hope that communication with the pharmacist is available and easy when we need it” patient 8 (Jazan)*

## Discussion

This study aimed to explore patient views, experiences factors contributing to DOACs safety from patients’ perspective. The factors recognised by participants in the study were categorised into three themes including factors affecting DOACs safety and strategies to promote DOACs safety. Our study findings showed that the discontinuation of DOACs due to non-availability of drug, suboptimal adherence, and lack of monitoring were key barriers to ensuring optimal use and patient safety. In addition, lack of information, and communication with physicians and pharmacists, inadequate education aware other factors associated with safe and effective use. Participants reported that improving patients’ awareness and knowledge, reinforcing education, enhancing accessibility to and communication with healthcare providers, using reminders techniques, in addition to intensifying follow-up activities are the perceived strategies to improve DOACs safety.

There is a dearth of studies looking into patient perspectives on their prescribed DOACs though it is very important to assess patients’ perspective and get them involved in initiatives to improve optimal DOACs use and their safety. To the best of our knowledge, this is the first study of its kind to examine patients’ perspectives in relation to with the use of DOACs using in depth qualitative analysis. A study conducted in 2014 by Sarrazin et al. to determine patients’ experience and perception regarding dabigatran using online discussion forums (468 posts), reported preference of dabigatran over other DOACs due to the lack of monitoring and dietary restrictions; however, the lack of an antidote was a safety concern [12].

The link between suboptimal adherence and safety concerns have been reported in previous studies [13]. Patients’ forgetfulness was found to be associated with DOACs medication incidents [13, 14]. Patient adherence to such medications is of paramount importance, as missing doses may lead to higher risks of thromboembolism [15].

Another important factor identified in this study was the availability of drugs. It is important to ensure seamless access to high-risk medications such as DOACs & their generics. Our patients’ concerns regarding drugs availability and the high cost that makes affordability and adherence challenging for patients are consistent with the findings from previous studies [15, 16].

Lack of adequate communication with healthcare professionals including pharmacies were particularly concerning. Patients in our study reported that lack of proper communication with healthcare providers was a key factor in compromising their safety. It is important to ensure optimal communications between patients and their healthcare professionals.

Recommendations to promote DOACs safety from patient perspectives identified in this study provides an opportunity to improve safe and effective use of DOACs. Regular follow-up in anticoagulation clinic and availability of health educators were examples of strategies to promote safety. Given that most patients who are users of DOACs are of advanced age, both patients and carers need to be involved, educated and communicate to promote safety as also recommended by previous studies [12].

### Strengths and limitations

Having patients actively participate in the decision-making process in their care is critical for improving the quality of care. Patients usually get overlooked and studies assessing their perspectives regarding this important health-related issue are lacking. Our study hence adds to the dearth in the literature. Despite data saturation being achieved, these results should be interpreted with care especially for generalisation purposes. Future studies could undertake a survey of patients utilising larger sample size with questionnaire tools developed based on the findings of this qualitative study.

Future studies could undertake a survey of patients utilising larger sample size with questionnaire tools developed based on the findings of this qualitative study.

## Conclusion

Patient knowledge regarding DOACs, optimal adherence, adequate communication between healthcare professionals and patients, timely access to clinics and the medicines are key factors that can promote optimal utilisation and safety of DOACs from patient point of view. There is a need to develop and evaluate theory-based interventions to promote patient knowledge, understanding and shared decision-making.

## Data Availability

All data generated or analysed during this study are included in this published article.

## Abbreviations

DOACs: Direct oral anticoagulants
NVAF: Non-valvular atrial fibrillation
HCP: Healthcare professionals
Covid-19: Coronavirus Disease 2019
COREQ: Consolidated Criteria for Reporting Qualitative Research
